# Circulating Tumor DNA as a Biomarker of Treatment Response and Minimal Residual Disease in Diffuse Large B-Cell Lymphoma: A Literature Review

**DOI:** 10.3390/jcm15145558

**Published:** 2026-07-15

**Authors:** Polina Chernova, Mariia Orlova, Elena Baryakh, Elena Misyurina, Tatiana Tolstykh, Ekaterina Zotina, Georgii Tyshkevich, Viktoriia Basova, Mira Suvorina, Andrey Misyurin, Marat Mingalimov

**Affiliations:** 1 Department of Hematology, I.M. Sechenov First Moscow State Medical University—Ministry of Health of Russia (Sechenov University), 119991 Moscow, Russia; chernova_p_s@student.sechenov.ru (P.C.); orlova_m_s@student.sechenov.ru (M.O.); baryakh_e_a@staff.sechenov.ru (E.B.); misyurina_e_n@staff.sechenov.ru (E.M.); tolstykh_t_n@staff.sechenov.ru (T.T.); zotina_e_n@staff.sechenov.ru (E.Z.); 2 Moscow Clinical Science and Research Center 52, 123182 Moscow, Russia; 3 FSBEI FPE «Russian Medical Academy of Continuous Professional Education» of the Ministry of Healthcare of the Russian Federation, 125993 Moscow, Russia; 4 N.I. Vavilov Institute of General Genetics, 119991 Moscow, Russia; vvvbasovavvv@gmail.com (V.B.); iogen@vigg.ru (M.S.); and@genetechnology.ru (A.M.); 5 FSBI FPE «Central State Medical Academy» of the Administrative Department of the President of the Russian Federation, 121359 Moscow, Russia; info@cgma.su

**Keywords:** DLBCL, ctDNA, sequencing, minimal residual disease, measurable residual disease, liquid biopsy, clonal hematopoiesis, fragmentomics, methylation, CAR-T, bispecific antibodies

## Abstract

Diffuse large B-cell lymphoma (DLBCL) is the most common subtype of aggressive non-Hodgkin lymphoma and is characterized by pronounced molecular heterogeneity that is not always fully captured by standard histopathological assessment. Circulating tumor DNA (ctDNA) is increasingly regarded as a promising liquid-biopsy biomarker that enables non-invasive molecular tumor profiling, assessment of tumor burden, dynamic monitoring of treatment response, and detection of measurable/minimal residual disease (MRD). Modern analytical platforms, ranging from PCR-based assays to next-generation sequencing approaches, including CAPP-Seq and PhasED-Seq, have substantially expanded the possibilities of molecular monitoring in DLBCL. This review summarizes current data on the biological characteristics of ctDNA, contemporary methods for its analysis, concordance between ctDNA and tumor-tissue mutational profiles, and the clinical significance of baseline ctDNA levels, early molecular response, post-treatment MRD status, and molecular surveillance during remission. Special attention is given to ctDNA monitoring in patients receiving novel immunotherapies, including CAR-T cell therapy, bispecific antibodies, and antibody–drug conjugates. Emerging multi-omic approaches integrating genomic, epigenomic, and fragmentomic data are discussed as promising future directions. Key limitations of clinical implementation include insufficient standardization of preanalytical and analytical workflows, the confounding effect of clonal hematopoiesis of indeterminate potential, variability across technological platforms, and the lack of completed prospective randomized interventional studies demonstrating improved outcomes when therapy is modified according to ctDNA status. Overall, ctDNA is currently a highly informative prognostic biomarker in DLBCL; however, its full implementation as a predictive tool for treatment selection requires further harmonization, prospective validation, and confirmation in interventional clinical trials.

## 1. Introduction

Diffuse large B-cell lymphoma (DLBCL) is a clinically and biologically heterogeneous group of aggressive non-Hodgkin lymphomas [1]. Although standard immunochemotherapy cures approximately 60–70% of patients, a substantial proportion develop primary refractory disease or relapse [1,2]. Treatment failure is driven, in part, by the complex molecular landscape of DLBCL, which includes genomic instability, diverse driver alterations, and clonal evolution under therapeutic selective pressure [3,4,5].

Conventional excisional lymph-node biopsy remains the diagnostic gold standard, but it provides a static immunomorphological and molecular snapshot of the tumor at a single time point [6,7]. This approach may be insufficient in the presence of spatial heterogeneity, where genetically distinct subclones coexist across different anatomical sites [8]. Early liquid-biopsy studies using immunoglobulin high-throughput sequencing demonstrated that non-invasive molecular monitoring in DLBCL is feasible [9]. At the same time, positron emission tomography combined with computed tomography (PET/CT), although central to response assessment, reflects metabolic activity rather than the molecular mechanisms of resistance or clonal dynamics [10].

In this context, liquid biopsy has created new opportunities for precision oncohematology [7,11]. Circulating tumor DNA (ctDNA), a fraction of cell-free DNA (cfDNA) released by tumor cells, represents a systemic molecular marker of tumor burden and clonal architecture [11,12]. ctDNA analysis enables minimally invasive detection of tumor-specific genetic alterations, molecular subtyping, dynamic treatment-response monitoring, minimal/measurable residual disease MRD assessment, and early detection of molecular relapse [13,14].

The aim of this review is to systematize current data on ctDNA biology in DLBCL, evaluate the evolution of analytical platforms from targeted sequencing to ultrasensitive phased-variant approaches, and summarize evidence supporting ctDNA as a biomarker of treatment response and MRD [11,15,16,17]. Particular attention is paid to the translational potential of ctDNA for overcoming spatial heterogeneity and enabling personalized response-adapted therapeutic strategies [8,13,14,18].

### Distinctive Contributions of the Present Review

The clinical utility of ctDNA in DLBCL has been addressed in several high-quality reviews [11,15,16,19,20,21]. However, important gaps remain. Previous reviews have focused primarily on biological principles, technological platforms, or general clinical applications, whereas fewer works have systematically distinguished prognostic from predictive utility, critically assessed methodological heterogeneity, or discussed ctDNA monitoring in the era of chimeric antigen receptor T-cell (CAR-T) therapy and bispecific antibodies (BsAbs).

The present review provides a clinically oriented, multi-omic synthesis integrating genomic, epigenomic, and fragmentomic ctDNA data [22,23,24,25,26]. We explicitly differentiate the well-established prognostic value of ctDNA from its still investigational predictive role and emphasize that ctDNA-guided therapeutic decisions have not yet been validated in completed prospective randomized interventional trials [19,27,28,29,30,31]. We also provide a critical appraisal of key studies, including sample size, study design, platform heterogeneity, clonal hematopoiesis of indeterminate potential (CHIP)-related confounding, and potential sources of bias [27,28,29,30,31,32,33,34,35,36,37]. Finally, we propose a conceptual framework for integrating longitudinal ctDNA monitoring with PET/CT across the DLBCL treatment continuum, including in patients receiving CAR-T cells, BsAbs, and antibody–drug conjugates (ADCs) [29,31,38,39,40,41,42,43,44,45,46,47,48,49].

## 2. Materials and Methods

### 2.1. Review Design and Reporting Standards

This is a clinically oriented narrative review with systematic components in the literature search and selection process. The review was conducted and reported in accordance with the Scale for the Assessment of Narrative Review Articles (SANRA) to improve methodological quality and transparency [50]. Elements of the PRISMA Extension for Scoping Reviews (PRISMA-ScR) were also applied to enhance transparency of study selection [51]. The PRISMA-ScR flow diagram is presented in Supplementary Figure S1.

### 2.2. Information Sources and Search Strategy

A comprehensive literature search was performed in PubMed/MEDLINE, Scopus, Web of Science Core Collection, and ClinicalTrials.gov from 1 January 2012 to 20 March 2026. The search was last updated on 20 March 2026. ClinicalTrials.gov entries and trial statuses were checked on the same date.

The primary Boolean search string used in PubMed/MEDLINE and adapted for each database was:

(“diffuse large B-cell lymphoma” OR “DLBCL” OR “large B-cell lymphoma” OR “LBCL”) AND (“circulating tumor DNA” OR “ctDNA” OR “liquid biopsy” OR “cell-free tumor DNA” OR “cfDNA”) AND (“minimal residual disease” OR “measurable residual disease” OR “MRD” OR “molecular monitoring” OR “treatment response” OR “CAPP-Seq” OR “PhasED-Seq” OR “phased variant” OR “clonal hematopoiesis”).

Manual searches of reference lists from included studies and recent reviews were also performed [11,15,16,19,20,21]. Conference abstracts from ASH, EHA, ASCO, and ICML were considered only when they presented pivotal data from large prospective studies not yet available as full-text publications; these are explicitly labeled as conference abstracts [49,52,53,54].

### 2.3. Eligibility Criteria

Inclusion criteria were:

1. peer-reviewed clinical studies, prospective or retrospective translational studies, systematic reviews, and meta-analyses involving adult patients with DLBCL or broader large B-cell lymphoma (LBCL) cohorts including DLBCL;
2. studies evaluating ctDNA for biological characterization, molecular profiling, concordance with tissue biopsy, prognostic or predictive value, treatment-response assessment, or MRD monitoring;
3. investigations using contemporary analytical platforms, including droplet digital PCR (ddPCR), immunoglobulin high-throughput sequencing (Ig-HTS), CAPP-Seq, circulating single-molecule amplification and re-sequencing technology (cSMART), PhasED-Seq, or other error-corrected targeted NGS assays [9,14,55,56,57,58,59,60,61].

Exclusion criteria were:

1. single case reports and very small case series;
2. non-peer-reviewed preprints;
3. pediatric lymphoma studies without adult DLBCL data;
4. editorials and commentaries without original data;
5. studies lacking sufficient methodological description of the ctDNA assay.

### 2.4. Study Selection and Data Extraction

Two independent reviewers screened titles and abstracts, followed by full-text assessment. Disagreements were resolved by consensus or consultation with a senior reviewer. Extracted data included the study design, cohort size, ctDNA platform, analytical sensitivity and specificity, sampling time points, clinical outcomes, and key limitations.

Because of substantial heterogeneity in technologies, sampling schedules, quantitative reporting, and clinical endpoints, quantitative meta-analysis was not performed. Instead, a narrative synthesis with critical methodological appraisal was conducted.

## 3. Results

### 3.1. Biological Characteristics of ctDNA

ctDNA is a tumor-derived fraction of cfDNA carrying genetic and epigenetic alterations characteristic of malignant cells [11,12,62]. cfDNA consists of short double-stranded DNA fragments circulating in plasma and generated mainly through apoptosis and necrosis. In healthy individuals, cfDNA is derived predominantly from hematopoietic cells, whereas in patients with cancer it represents a mixture of normal and tumor-derived DNA [11,12,62].

Physiological and pathological factors such as inflammation, trauma, physical activity, infection, and treatment-related tissue injury may increase the amount of non-tumor cfDNA, thereby reducing the relative ctDNA fraction and complicating detection [11,12,62,63]. The ctDNA fraction may vary widely between patients and disease states, reflecting tumor burden, anatomical distribution, vascular access, cell turnover, and biological features of the lymphoma.

A key feature of cfDNA is its fragmented structure. The dominant fragment length in healthy individuals is approximately 167 bp, corresponding to nucleosome-associated DNA [11,25,64]. Tumor-derived fragments are often shorter, with a peak around 145 bp, reflecting altered chromatin structure, aberrant methylation, and increased susceptibility to nuclease degradation [12,25,65]. Fragmentomic features, including fragment size, end motifs, and nucleosome positioning, may therefore provide additional information beyond mutation detection [24,25,26,65,66].

ctDNA is released into the circulation through apoptosis, necrosis, and active secretion within extracellular vesicles [12,67,68]. In aggressive lymphomas, high proliferative activity and spontaneous tumor-cell death often result in higher ctDNA fractions than those observed in many solid tumors [11,14,16]. Antitumor therapy may transiently increase ctDNA levels through rapid tumor-cell lysis, followed by clearance in responding patients. Because ctDNA has a short half-life, generally estimated in minutes to a few hours, plasma ctDNA levels may reflect real-time tumor dynamics rather than cumulative tumor burden [62,63].

These biological properties make DLBCL a particularly informative model for liquid-biopsy applications. However, findings from solid tumors or pediatric malignancies should not be extrapolated directly to adult DLBCL without disease-specific validation [10,11,16,69].

**Figure 1:**
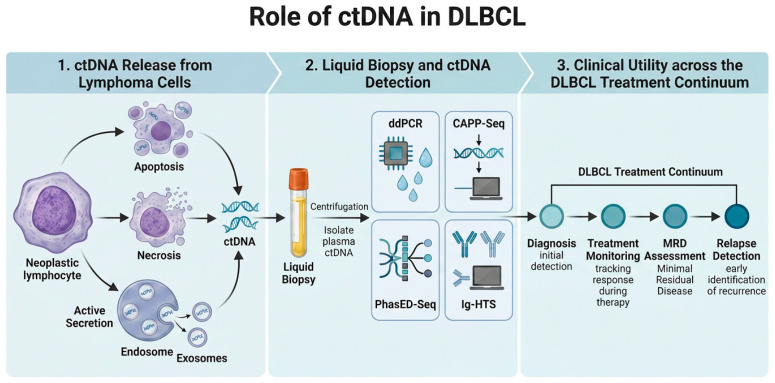
Schematic overview of ctDNA release, detection, and clinical applications in DLBCL. The figure illustrates ctDNA release from tumor cells through apoptosis, necrosis, and extracellular vesicles; liquid-biopsy workflow including blood collection, plasma separation, cfDNA extraction, and ctDNA detection; and clinical applications including baseline molecular profiling, treatment-response monitoring, MRD assessment, and relapse detection.

The key steps of ctDNA release, detection, and clinical applications along the DLBCL treatment continuum are summarized schematically in Figure 1.

### 3.2. ctDNA Detection

The spectrum of ctDNA detection methods in DLBCL ranges from single-locus polymerase chain reaction (PCR)-based assays to multiplex next-generation sequencing (NGS) platforms [11,15,16,38]. Platform selection depends on the intended application: detection of predefined variants, broad molecular profiling, MRD monitoring, or relapse surveillance.

Strict preanalytical standardization is essential. Plasma is preferred over serum because serum carries a higher risk of genomic DNA contamination due to leukocyte lysis during clotting [11,38,42,43,44]. Blood collected in K2EDTA tubes should generally be processed within approximately 6 h, whereas cfDNA-stabilizing tubes may preserve samples for several days depending on manufacturer specifications [38,42,43,44].

Historically, allele-specific PCR and immunoglobulin gene rearrangement assays were central to MRD detection [9,59]. Droplet digital PCR (ddPCR) improved absolute quantification and analytical sensitivity for predefined recurrent mutations, such as MYD88 L265P, but remains limited by low multiplexing capacity and the need for prior knowledge of the target alteration [57,58].

Immunoglobulin high-throughput sequencing (Ig-HTS) identifies patient-specific clonal V(D)J rearrangements and enables sensitive MRD monitoring [9,59,60]. However, in DLBCL, somatic hypermutation may affect primer-binding regions, preventing identification of a trackable clonotype in a subset of patients [9,11,15].

Hybrid-capture NGS approaches, including Cancer Personalized Profiling by deep Sequencing (CAPP-Seq), enable broad targeted profiling of recurrently mutated lymphoma genes [14,55,61]. Integrated digital error suppression improves analytical sensitivity and reduces sequencing noise [55,61]. Circulating single-molecule amplification and re-sequencing technology (cSMART), a single-molecule barcoding approach, has also been applied to DLBCL plasma genotyping, although clinical validation remains more limited [56,70,71].

Phased Variant Enrichment and Detection Sequencing (PhasED-Seq) represents one of the most sensitive ctDNA-MRD approaches. It detects phased variants—two or more somatic variants located in cis on the same DNA molecule—which are enriched in B-cell lymphomas due to the aberrant activity of activation-induced cytidine deaminase (AID) [17]. By requiring linked variants, PhasED-Seq substantially reduces background noise and improves MRD detection at extremely low ctDNA fractions [17,29].

ctDNA assays may be classified as tumor-informed or tumor-agnostic (Table 1). Tumor-informed assays track patient-specific variants identified from baseline tumor tissue or high-burden pretreatment plasma, whereas tumor-agnostic assays use fixed panels targeting recurrently altered genes [11,15,16,38,72] (Table 2).

### 3.3. Concordance Between ctDNA and Tumor Biopsy Mutational Profiles

Studies comparing ctDNA and tumor-tissue profiles show that plasma ctDNA can reproduce key molecular features of DLBCL while also detecting additional alterations not captured by single-site biopsy [13,14,18,32,33].

In a paired tissue-plasma study of newly diagnosed DLBCL, Song et al. reported substantial mutation-level overlap between baseline ctDNA and matched tumor tissue after filtering germline and CHIP-associated variants [32]. Importantly, ctDNA also detected mutations present only in plasma, supporting its ability to capture spatially heterogeneous subclones.

ctDNA-based molecular classification has also shown strong concordance with tissue-based approaches. Moia et al. demonstrated high concordance between ctDNA and tumor material for LymphGen molecular subtype assignment [22]. In the POLARIX translational program, reported as a conference abstract, ctDNA profiling identified molecular subtypes in cases that were unclassifiable using tissue sequencing alone [52]. Xia et al. similarly showed that ctDNA improved mutation detection and enabled additional LymphGen subtype assignment in high-risk DLBCL [33].

Discordant ctDNA/tissue findings require careful interpretation. ctDNA-positive/tissue-negative discordance may reflect spatial heterogeneity or sampling limitations of tissue biopsy. Conversely, ctDNA-negative/tissue-positive discordance may occur in low-shedding tumors, low-volume disease, or anatomically restricted disease sites [8,11,16,33]. Thus, ctDNA should be viewed as complementary to tissue biopsy rather than a universal replacement.

### 3.4. Clinical Significance of ctDNA in DLBCL

#### 3.4.1. Baseline ctDNA as a Marker of Tumor Burden and Prognosis

Before ctDNA can be used for response monitoring, baseline quantitative characteristics should be established. Baseline ctDNA concentration correlates with tumor burden, stage, lactate dehydrogenase (LDH) level, International Prognostic Index (IPI)/Revised International Prognostic Index (R-IPI), B symptoms, and metabolic tumor volume [14,27,34].

Kurtz et al. demonstrated that elevated baseline ctDNA measured by CAPP-Seq was associated with inferior event-free survival (EFS) in both first-line and relapsed settings, independently of IPI, molecular subtype, and TMTV [14]. Li et al. similarly reported that high baseline ctDNA levels were associated with inferior progression-free survival (PFS) and overall survival (OS), and remained an independent prognostic factor in multivariable analysis [27]. Narkhede et al. confirmed that baseline ctDNA correlated with advanced stage and R-IPI, although survival associations were limited by small cohort size [34]. A systematic review and meta-analysis further supported the adverse prognostic value of elevated baseline ctDNA in DLBCL [35].

These data support baseline ctDNA as a robust marker of tumor burden. However, thresholds defining a “high” ctDNA burden remain platform-specific and are not yet standardized across assays [11,15,38,42,43,44].

#### 3.4.2. Early Molecular Response During Therapy

Dynamic ctDNA reduction during immunochemotherapy provides early information on treatment response. Kurtz et al. introduced early molecular response (EMR), defined as a ≥2-log ctDNA reduction after one treatment cycle, and major molecular response (MMR), defined as a ≥2.5-log reduction after two cycles [14]. Both metrics were strongly associated with improved outcomes.

Subsequent studies confirmed the prognostic value of early ctDNA clearance. Li et al. showed that molecular response was associated with superior PFS and OS [27]. Alcoceba et al. demonstrated that major molecular response after two induction cycles was associated with markedly improved 2-year PFS [28]. Roschewski et al. later integrated data from five prospective LBCL studies and showed that ctDNA clearance after two cycles and at the end of therapy strongly stratified relapse risk [29]. Because this cohort included DLBCL and related aggressive LBCL entities, the findings should be interpreted as DLBCL-relevant but not DLBCL-exclusive [29].

#### 3.4.3. End-of-Treatment MRD

Post-treatment ctDNA-MRD status is one of the strongest molecular markers of residual disease persistence [29,31,36,37]. Roschewski et al. showed that end-of-treatment ctDNA positivity identified patients with a markedly increased risk of relapse [29]. Wang et al. prospectively validated the prognostic value of ctDNA-MRD after first-line therapy in LBCL, demonstrating that molecular MRD adds information beyond PET/CT response assessment [31] (Table 3).

Soscia et al. demonstrated that MRD assessment using immunoglobulin rearrangements in ctDNA stratified patients by progression risk and refined prognosis among patients with partial response on imaging [36]. Meta-analytic data reported in conference-abstract form further support the association between post-treatment ctDNA persistence and inferior PFS, including among patients with complete metabolic response on PET/CT [53,54].

#### 3.4.4. Molecular Surveillance During Remission

Serial ctDNA monitoring during remission may detect molecular relapse before clinical or radiological progression. Roschewski et al. demonstrated that ctDNA detected relapse a median of 3.5 months before conventional assessment, with a high positive predictive value (PPV) and a negative predictive value (NPV) [37]. Scherer et al. showed that longitudinal ctDNA profiling can reveal clonal evolution and emergence of resistance-associated alterations [18].

Although these findings support preclinical relapse detection, the optimal frequency and duration of surveillance, and the benefit of pre-emptive therapy based solely on molecular relapse, remain unresolved [19,30] (Table 4).

### 3.5. Epigenomic and Fragmentomic Features of ctDNA and Multi-Omic Integration

Although mutation-based profiling remains the most established ctDNA approach in DLBCL, epigenomic and fragmentomic characteristics provide complementary biological information [22,23,24,25,26].

#### 3.5.1. DNA Methylation Profiling

DNA methylation is one of the earliest and most stable epigenetic alterations in cancer. Tumor cells exhibit genome-wide hypomethylation and focal hypermethylation of CpG islands, generating tumor-type-specific signatures that may be preserved in ctDNA [23,24,65].

Methylation-based cfDNA analysis can assist tissue-of-origin inference and may help distinguish tumor-derived fragments from hematopoietic or inflammatory background cfDNA [23,24]. In DLBCL, methylation-based ctDNA assays remain less mature than mutation-based methods, but they are promising for biological classification and sensitivity enhancement when integrated with genomic data [20,22,23,24].

#### 3.5.2. Fragmentomic Analysis

Fragmentomics evaluates cfDNA fragment length, end motifs, and nucleosome-positioning patterns [25,26,66]. Tumor-derived ctDNA fragments are typically shorter than hematopoietic cfDNA fragments, and genome-wide fragmentation patterns may support cancer detection and tissue-of-origin inference [25].

Nucleosome footprinting and cfDNA fragmentation profiles can also infer gene-expression programs without direct RNA sequencing [26,66]. In DLBCL, such approaches could potentially support non-invasive cell-of-origin classification and identification of active oncogenic pathways, although this remains investigational.

#### 3.5.3. Multi-Omic Integration

The greatest future potential may lie in integrating genomic, epigenomic, and fragmentomic features from the same plasma sample [22,23]. Machine learning approaches combining orthogonal ctDNA layers may improve detection at low tumor fractions and reduce false-positive mutation calls related to CHIP. However, prospective DLBCL-specific validation is still required [20,22,23,39,40,41,42].

### 3.6. Critical Appraisal of Methodological Quality

The evidence supporting ctDNA as a prognostic biomarker in DLBCL is strong but methodologically heterogeneous [11,15,16,19,20]. Several studies are limited by small sample size, including cohorts with fewer than 75 patients [27,28,32,34,36]. Small cohorts reduce statistical power, limit subgroup analyses, and increase uncertainty around hazard ratio (HR) estimates.

Study designs vary from prospective to retrospective and mixed designs, creating differences in selection bias and generalizability [14,22,27,28,29,30,31,32,33,34,35,36,37]. Platform heterogeneity also limits direct comparison. CAPP-Seq, Ig-HTS, custom targeted NGS, Signatera, cSMART, and PhasED-Seq differ in target design, quantification, error correction, and reporting units [9,14,17,29,34,55,56,57,58,59,60,61].

CHIP filtering was not uniformly performed, especially in earlier studies. This is clinically important because CHIP-associated variants may mimic tumor-derived mutations, particularly at low variant allele frequencies (VAFs) [39,40,41,42]. Assays based on immunoglobulin rearrangements or phased variants are less susceptible to CHIP-associated single-variant noise, but mutation-based panels require matched leukocyte sequencing or rigorous filtering strategies [17,36,37,39,40,41,42].

Finally, several influential datasets remain available only as conference abstracts [49,52,53,54]. Their findings should therefore be interpreted cautiously until full peer-reviewed publications are available (Table 5).

### 3.7. ctDNA Monitoring in the Era of CAR-T Cell Therapy and Bispecific Antibodies

The treatment landscape of relapsed/refractory DLBCL within the broader LBCL category has changed substantially with CAR-T cell therapy, bispecific antibodies, and antibody–drug conjugates [45,46,47,48]. These modalities produce distinct biological kinetics, creating new opportunities and challenges for ctDNA monitoring.

#### 3.7.1. CAR-T Cell Therapy

CAR-T cell therapy may induce rapid tumor lysis, which can be reflected by an early transient ctDNA increase followed by rapid clearance in responders [45,46]. Persistent or rising ctDNA after infusion has been associated with primary resistance or early relapse.

Frank et al. showed that ctDNA clearance by day 28 after axicabtagene ciloleucel was associated with superior PFS in patients with LBCL [45]. ctDNA also refined prognosis among patients with complete metabolic response on PET/CT, suggesting that molecular assessment may detect residual disease below imaging sensitivity.

In the CAR-T setting, CHIP is particularly relevant because lymphodepleting conditioning may alter hematopoietic clonal dynamics. Therefore, lymphoma-specific markers and matched leukocyte sequencing are especially important when interpreting post-CAR-T ctDNA results [39,40,41,42,45].

#### 3.7.2. Bispecific Antibodies

CD20 × CD3 bispecific antibodies, including glofitamab and epcoritamab, have demonstrated clinically meaningful activity in relapsed/refractory DLBCL and LBCL [47,48]. Unlike CAR-T cells, these agents are administered repeatedly, and ctDNA clearance kinetics may be more gradual.

ctDNA monitoring could potentially inform treatment duration, identify deep molecular remissions, and detect early resistance. However, ctDNA-guided discontinuation, escalation, or consolidation strategies during bispecific-antibody therapy have not yet been prospectively validated [19,30,47,48].

#### 3.7.3. Antibody–Drug Conjugates

Antibody–drug conjugates (ADCs), including polatuzumab vedotin and loncastuximab tesirine, are increasingly used in DLBCL treatment sequences. ctDNA kinetics during ADC therapy remain less well characterized than in CAR-T or bispecific antibody settings [16]. Translational data from the POLARIX program suggest that ctDNA may support molecular profiling, but detailed kinetic validation remains limited [52].

## 4. Limitations and Future Perspectives

### 4.1. Prognostic Versus Predictive Utility

A key distinction must be made between prognostic and predictive biomarkers. A prognostic biomarker provides information about outcome regardless of treatment, whereas a predictive biomarker identifies patients likely to benefit from a specific therapeutic intervention [19].

Current evidence strongly supports ctDNA as a prognostic biomarker in DLBCL. Elevated baseline levels, failure to achieve early molecular response, and post-treatment MRD positivity are consistently associated with inferior outcomes [14,27,28,29,30,31,32,33,34,35,36,37]. However, ctDNA has not yet been validated as a predictive biomarker in the strict sense. No completed prospective randomized trial has demonstrated that changing treatment based on ctDNA status improves outcomes compared with standard management [19,30].

The ongoing SHORTEN ctDNA trial is one example of an interventional effort designed to test ctDNA-guided therapy optimization [30]. Until such data are available, ctDNA-directed treatment modifications should be considered investigational.

### 4.2. Standardization of the Analytical Workflow

Preanalytical variables, including blood tube type, time to centrifugation, centrifugation protocol, cfDNA extraction method, and storage conditions, strongly influence ctDNA results [38,42,43,44]. Analytical variability arises from differences in panel design, sequencing depth, error suppression, variant calling, CHIP filtering, and reporting units [11,15,38,42].

Universal thresholds for high ctDNA burden, molecular response, and MRD positivity have not yet been established. Harmonized reporting should include assay type, cfDNA input, limit of detection, tracked variants, CHIP-filtering method, quantitative unit, and interpretive limitations [38,42].

### 4.3. Clonal Hematopoiesis of Indeterminate Potential

CHIP is one of the most clinically significant sources of biological false-positive results in ctDNA analysis [11,39,40,41,42,76]. CHIP-associated variants commonly affect genes such as DNMT3A, TET2, ASXL1, PPM1D, SF3B1, and TP53, which may overlap with genes included in lymphoma ctDNA panels [39,40,41,42].

Inadequate CHIP filtering can lead to false-positive MRD results, overestimation of baseline ctDNA burden, or misinterpretation of molecular relapse. Matched leukocyte sequencing is therefore the preferred approach for mutation-based ctDNA assays [41,42]. When matched leukocyte DNA is unavailable, gene-level filtering, VAF dynamics, and longitudinal interpretation may reduce but not eliminate this risk.

Lymphoma-specific markers, including clonal immunoglobulin rearrangements and phased variants, are less susceptible to CHIP-associated single-variant background noise [17,36,37].

### 4.4. Harmonization Initiatives

Several international initiatives have developed general frameworks for cfDNA and ctDNA assay validation. The Association for Molecular Pathology (AMP)/College of American Pathologists (CAP) recommendations provide guidance on analytical validation, limit-of-detection reporting, and quality control [42]. BloodPAC has proposed generic protocols for analytical validation of NGS-based ctDNA assays [43]. European liquid-biopsy initiatives and the Foundation for the National Institutes of Health (FNIH) have supported harmonization, reference materials, and quality assessment frameworks [77,78].

Lymphoma-specific standardization remains less mature. Key future requirements include:

1. harmonized preanalytical protocols;
2. certified reference materials;
3. consensus CHIP-filtering strategies;
4. standardized reporting units and MRD definitions;
5. external quality assessment (EQA) programs;
6. prospective interventional validation of ctDNA-guided treatment strategies.

## 5. Conclusions

ctDNA has established a new paradigm of molecular monitoring in DLBCL, enabling non-invasive genotyping, dynamic assessment of tumor burden, early evaluation of treatment response, and highly sensitive MRD detection [11,13,14,15,16,17,27,28,29,30,31,36,37]. Baseline ctDNA level, early molecular response, and post-treatment MRD status consistently provide clinically meaningful prognostic information.

Emerging multi-omic strategies integrating genomic, epigenomic, and fragmentomic data may further improve sensitivity, biological interpretation, and molecular subtyping [22,23,24,25,26]. ctDNA monitoring in patients receiving CAR-T cell therapy, bispecific antibodies, and antibody–drug conjugates is a rapidly evolving field, although most available data remain translational or observational [45,46,47,48].

At present, ctDNA should be regarded primarily as a prognostic biomarker. Its predictive role—namely, the ability to guide treatment selection or modification and improve clinical outcomes—has not yet been confirmed in completed prospective randomized interventional trials [19,30]. Therefore, ctDNA-guided treatment decisions should remain investigational until validated in appropriately designed clinical studies.

## Figures and Tables

**Table 1:** Comparative characteristics of tumor-informed and tumor-agnostic ctDNA approaches.

Characteristic	Tumor-Informed Approach	Tumor-Agnostic Approach
Principle	Personalized assay based on patient-specific variants	Fixed panel targeting recurrently altered genes
Baseline material	Usually required	Not required for initial profiling
Strengths	High specificity; optimal for MRD	Scalable; useful for de novo genotyping
Limitations	Requires baseline material and assay customization	Higher background noise; CHIP filtering required
Best application	MRD monitoring and relapse detection	Initial molecular profiling and dynamic monitoring

**Table 2:** (**a**) Technical characteristics of ctDNA detection methods in DLBCL. (**b**) Clinical validation and practical applications of ctDNA detection methods in DLBCL.

**(a)**				
**Method**	**Analytical Sensitivity**	**Analytical Specificity**	**Advantages**	**Limitations**
ddPCR	approximately 10^−4^	High	Absolute quantitative assessment; cost-effectiveness; monitoring of known recurrent mutations (MYD88 L265P, etc.)	Low multiplexing capacity (single loci); inability to perform comprehensive genotyping; high sensitivity to contamination
Ig-HTS	approximately 10^−6^	High	Standardized monitoring of V(D)J rearrangements; biologically independent of CHIP	Requires baseline tumor material to identify a clonal marker; the clonal reporter is not identified in approximately 20% of patients due to somatic hypermutation; low cfDNA input may reduce analytical efficiency
CAPP-Seq	approximately 2 × 10^−5^ (with iDES)	High	Multiplex analysis; simultaneous molecular profiling and assessment of ctDNA/MRD kinetics; integration of iDES minimizes sequencing background noise	Laboratory workflow and bioinformatic analysis are labor-intensive; when ultra-low VAFs are detected, preanalytical quality and complex filtering of biological background noise are critical
cSMART	approximately 10^−6^	High	Single-molecule barcoding based on NGS; well suited for non-invasive plasma genotyping; originally validated in solid tumors, subsequently applied in DLBCL [29]	Limited clinical validation and absence of standardized protocols for use in DLBCL
PhasED-Seq	approximately 5 × 10^−7^	High	Detection of linked (phased) variants providing ultrahigh sensitivity; biologically independent of CHIP	Requires panel customization and baseline tumor material to identify phased variants; bioinformatic analysis and laboratory logistics are highly complex
(**b**)				
**Method**	**Level of Clinical Validation**		**Optimal Application**	**Key Practical Considerations**
ddPCR	Limited clinical validation in DLBCL; used exclusively for targeted monitoring of previously identified molecular targets		Monitoring of known recurrent mutations (MYD88 L265P, etc.)	Cost-effective for single-locus monitoring; requires prior knowledge of mutation; limited by tumor heterogeneity
Ig-HTS	Moderate clinical validation in DLBCL without broad implementation in practice		MRD monitoring using clonal immunoglobulin rearrangements	Biologically independent of CHIP; clonal reporter not identified in ~20% of DLBCL patients; FDA-approved for other hematologic malignancies
CAPP-Seq	High validation for prognosis assessment and dynamic monitoring in DLBCL, but not yet integrated into routine clinical standards		Initial genotyping; dynamic monitoring; molecular subtyping	Broad gene coverage; iDES reduces background noise; requires specialized bioinformatic expertise
cSMART	Limited clinical validation in DLBCL; used mainly within research protocols		Non-invasive plasma genotyping; research applications	High single-molecule accuracy; validated primarily in solid tumors; one DLBCL study published [29]
PhasED-Seq	High clinical validity in DLBCL with inclusion in NCCN recommendations		Ultrasensitive MRD monitoring; early relapse detection	Ultrahigh sensitivity (5 × 10^−7^); biologically independent of CHIP; requires baseline tumor tissue for phased variant identification

**Table 3:** Conceptual integration of PET/CT and ctDNA-MRD after completion of therapy. This framework is conceptual and should not be used for treatment decisions outside clinical trials or guideline-supported contexts [29,31,73,74,75].

PET/CT	ctDNA-MRD	Interpretation	Potential Management Approach
Negative	Negative	Deep molecular-metabolic remission	Standard guideline-directed follow-up
Positive	Negative	Possible false-positive metabolic activity	Repeat imaging or biopsy if clinically indicated
Negative	Positive	Molecular residual disease despite metabolic remission	Intensified monitoring; clinical-trial enrollment
Positive	Positive	Concordant evidence of residual disease	Histologic confirmation when feasible; management as R/R DLBCL

**Table 4:** Key clinical studies evaluating ctDNA in DLBCL/LBCL.

Study	Design	N	Platform	Main Finding
Kurtz et al. 2018 [14]	Prospective cohorts	217	CAPP-Seq	Baseline ctDNA and early molecular response predicted outcomes
Li et al. 2022 [27]	Real-world cohort	52	Targeted NGS	Baseline ctDNA and clearance predicted PFS/OS
Narkhede et al. 2024 [34]	Retrospective	41	Signatera	Clearance associated with improved EFS/OS
Alcoceba et al. 2024 [28]	Prospective	44	Targeted NGS	Major molecular response after two cycles predicted PFS
Roschewski et al. 2025 [29]	Integrative prospective analysis	137	PhasED-Seq	End-of-treatment MRD strongly predicted relapse risk
Soscia et al. 2025 [36]	Retrospective	73	Ig-HTS	MRD stratified PFS and refined imaging-based prognosis
Wang et al. 2026 [31]	Prospective validation	LBCL cohort	ctDNA-MRD	Post-treatment MRD added prognostic value to PET/CT
Roschewski et al. 2015 [37]	Prospective surveillance	126	Ig-HTS	Molecular relapse preceded clinical relapse

**Table 5:** Critical appraisal of selected ctDNA studies in DLBCL/LBCL.

Study	Strengths	Main Limitations
Kurtz et al. 2018 [14]	Large cohort; serial sampling; validation cohort	Platform-specific thresholds; heterogeneous settings
Roschewski et al. 2015 [37]	Longitudinal surveillance; lead time over imaging	Serum samples; clonotype identification limitations
Song et al. 2025 [32]	Paired tissue-plasma analysis; CHIP/germline filtering	Small single-center cohort
Li et al. 2022 [27]	Real-world data; survival correlations	Small cohort; custom panel
Alcoceba et al. 2024 [28]	Prospective design; structural variants included	Few non-responders; specialized workflow
Xia et al. 2024 [33]	Improved mutation detection and LymphGen classification	Limited follow-up; single platform
Moia et al. 2025 [22]	Multicenter molecular clustering	Retrospective component
Roschewski et al. 2025 [29]	Prospective datasets; PhasED-Seq; PET/CT comparison	LBCL rather than DLBCL-exclusive cohort
Narkhede et al. 2024 [34]	Tumor-informed commercial assay	Very small sample size
Soscia et al. 2025 [36]	Clinically relevant MRD time points	Retrospective design

## Data Availability

No new data were created or analyzed in this study. Data sharing is not applicable to this article.

## Supplementary Materials

The following supporting information can be downloaded at: https://www.mdpi.com/article/10.3390/jcm15145558/s1, Figure S1: PRISMA-ScR flow diagram of the study selection process.

## Abbreviations

The following abbreviations are used in this manuscript:

ADC	antibody–drug conjugate
AID	activation-induced cytidine deaminase
AMP	Association for Molecular Pathology
BCT	blood collection tube
bp	base pairs
BsAb	bispecific antibody
CAP	College of American Pathologists
CAPP-Seq	Cancer Personalized Profiling by deep Sequencing
CAR-T	chimeric antigen receptor T-cell
cfDNA	cell-free DNA
cfMeDIP-seq	cell-free methylated DNA immunoprecipitation and sequencing
CHIP	clonal hematopoiesis of indeterminate potential
CI	confidence interval
CRS	cytokine release syndrome
cSMART	circulating single-molecule amplification and re-sequencing technology
ctDNA	circulating tumor DNA
ddPCR	droplet digital polymerase chain reaction
DLBCL	diffuse large B-cell lymphoma
EFS	event-free survival
EMR	early molecular response
FDA	United States Food and Drug Administration
hGE/mL	haploid genome equivalents per milliliter
ICANS	immune effector cell-associated neurotoxicity syndrome
iDES	integrated digital error suppression
Ig-HTS	immunoglobulin high-throughput sequencing
IPI	International Prognostic Index
LBCL	large B-cell lymphoma
LDH	lactate dehydrogenase
LOD	limit of detection
MMR	major molecular response
MRD	minimal/measurable residual disease
NCCN	National Comprehensive Cancer Network
NGS	next-generation sequencing
NPV	negative predictive value
OS	overall survival
PCR	polymerase chain reaction
PET/CT	positron emission tomography/computed tomography
PFS	progression-free survival
PPV	positive predictive value
R/R	relapsed/refractory
R-IPI	Revised International Prognostic Index
SNV	single-nucleotide variant
TMTV	total metabolic tumor volume
VAF	variant allele frequency
V(D)J	variable, diversity, and joining gene segment
