# Supplementary figures and images for: Circulating Tumor DNA as a Biomarker of Treatment Response and Minimal Residual Disease in Diffuse Large B-Cell Lymphoma: A Literature Review
Source: J Clin Med. 2026 Jul 15;15(14):5558. doi: 10.3390/jcm15145558 (PMC13412977; doi:10.3390/jcm15145558)

Figure S1: PRISMA-ScR flow diagram of the study selection process.

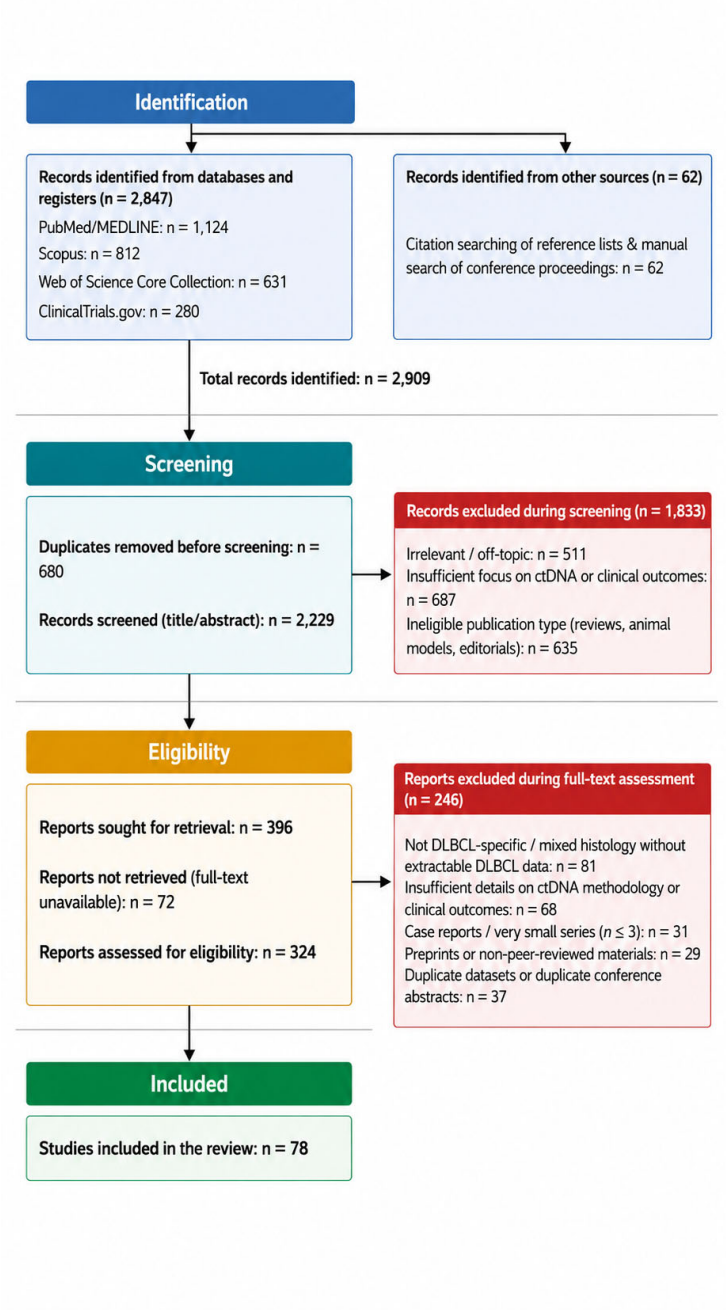

Supplement: Supplementary file 1 [file jcm-15-05558-s001.zip › jcm-4427824-supplementary.pdf]
